# Supplementary material for: The Impact of Sex, Body Mass Index, Age, Exercise Type and Exercise Duration on Interstitial Glucose Levels during Exercise
Source: Sensors (Basel). 2023 Nov 9;23(22):9059. doi: 10.3390/s23229059 (PMC10674905; doi:10.3390/s23229059)
Supplement: Supplementary file 1 [file sensors-23-09059-s001.zip › sensors-2617180-supplementary.pdf]

Supplementary Table S1: Participant characteristics within each subgroup analyses.

Variable: sex

	males	females
N	44	77
# of exercise events	228	435
Age, yrs (mean ± SD)	23.9 ± 19	32.4 ± 19.8
BMI, kg/m^2 (mean ± SD)	21.3 ± 4.4	22.1 ± 3.9

Variable: BMI Category

BMI Category (kg/m^2)	Underweight (<18.5 kg/m^2)	Normal Weight (19-24.9 kg/m^2)	Overweight (25-29.9 kg/m^2)
Total N (# of females)	31 (12)	62 (49)	29 (17)
# of exercise events	173	327	139
Age, yrs (mean ± SD)	11.4 ± 4.7	32.4 ± 19.9	41.8 ± 16.8
BMI (mean ± SD)	16.8 ± 1.5	21.7 ± 1.7	27.5 ± 1.6

Variable: Age Category

Age Category (years)	6-19	20-39	40-59	60-89
Total N (% female)	51 (26)	35 (24)	19 (16)	17 (12)
# of exercise events	276	156	110	121
Age, yrs (mean ± SD)	12 ± 3.2	26.8 ± 6	47.3 ± 6.1	66.2 ± 6
BMI, kg/m^2 (mean ± SD)	18.5 ± 2.6	23.9 ± 3.3	24.7 ± 3.4	24.4 ± 2.9

Variable: Exercise Type

Exercise type	Aerobic	Resistance	Mixed
Total N (% female)	115 (76)	30 (18)	20 (18)
# of exercise events	534	51	39
Age, yrs (mean ± SD)	29.1 ± 19.9	34.9 ± 19	39.1 ± 19.1
BMI, kg/m^2 (mean ± SD)	21.7 ± 4.1	22.7 ± 3.8	23.1 ± 3.5

Variable: Exercise Duration

Exercise Duration (min)	<20	21-39	40-59	60-89	90-119	120-300
Total N (# of females)	44 (66%)	75 (71%)	55 (56%)	73 (66%)	27 (56%)	33 (15)
# of exercise events	105	179	117	161	46	55
Age, yrs (mean ± SD)	30.1 ± 21.8	30.3 ± 20.2	31.7 ± 20.6	31.4 ± 20.2	28.9 ± 18.6	20.3 ± 14.8
BMI, kg/m^2 (mean ± SD)	22 ± 4.2	21.7 ± 4.3	22.4 ± 4.2	21.7 ± 4	21.9 ± 4	19.6 ± 3.2
